# Macrophylloflavone: A New Biflavonoid from *Garcinia macrophylla* Mart. (Clusiaceae) for Antibacterial, Antioxidant, and Anti-Type 2 Diabetes Mellitus Activities

**DOI:** 10.1155/2020/2983129

**Published:** 2020-05-12

**Authors:** Hawa Purnama Celala Ary Cane, Nurdin Saidi, Mustanir Yahya, Darusman Darusman, Erlidawati Erlidawati, Safrida Safrida, Musri Musman

**Affiliations:** ^1^Graduate School of Mathematics and Applied Sciences, Universitas Syiah Kuala, Banda Aceh 23111, Indonesia; ^2^Department of Chemistry, Universitas Syiah Kuala, Banda Aceh 23111, Indonesia; ^3^Department of Soil Science, Universitas Syiah Kuala, Banda Aceh 23111, Indonesia; ^4^Department of Chemistry Education, Universitas Syiah Kuala, Banda Aceh 23111, Indonesia; ^5^Department of Biology Education, Universitas Syiah Kuala, Banda Aceh 23111, Indonesia

## Abstract

Investigations of antibacterial, antioxidant, and anti-type 2 diabetes mellitus activities have been carried out on *Garcinia macrophylla* Mart. plant extract fractions. An isolate from a fraction of ethyl acetate extract was characterized with spectroscopic data. A new biflavonoid compound was found to have a skeleton of 5,7,4′,5″,7″,3‴,4‴-heptahydroxyflavanone[3-6″] flavones which was named macrophylloflavone (**1**). The compound was evaluated for its antibacterial activity against *Escherichia coli* ATCC 25922 and *Staphylococcus aureus* ATCC 25923 with cephazolin as a positive control, antioxidant assay against 2,2 diphenyl-1-picrylhydrazyl (DPPH) with ascorbic acid as the positive control, and anti-type 2 diabetes mellitus treatment with metformin as a positive control. The biflavonoid compound exhibited a good inhibition for bacteria and free radical DPPH. Furthermore, biflavonoid compound treatment on the diabetic rats suggested its ability to decrease the blood glucose level. This study provided evidence that the plant has antibacterial, antioxidant, and antidiabetic properties.

## 1. Introduction

Degenerative diseases contribute the most number of world's mortalities and morbidities. According to many studies, the main factor causing the degenerative diseases is free radicals, which are actively produced through human body metabolic process [1–4]. Diabetes mellitus is an example of degenerative diseases which is infamous for the death of 1.6 million people worldwide in 2016. The disease is believed to be the main cause due to a lack of insulin secretion by the pancreas or metabolic disorders when blood glucose levels are higher (hyperglycemia) than normal levels [5]. Recent studies also found that people with the disease are more susceptible to dermatologic infections, such as staphylococcal follicular skin infections, erysipelas, and cellulitis. This is made worse by the fact that the human body is vulnerable to pathogenic bacteria, even when we carry out our daily routine [6, 7]. It leads to a situation where patients take many kinds of medicines. To deal with the bacterial disease problems, many people rely on antibiotics. However, the rise of antibiotic-resistant bacteria has grown a concern to find the alternative for antibiotics [8]. In addition, synthetic drugs are often used for maintenance-free radical and diabetes mellitus, not to mention the fact that synthetic drug intake can exert multiple adverse effects on health [9, 10].

As an alternative, this study proposed the use of ethnomedicinal plants which have antibacterial, antioxidant, and antidiabetic activities [11, 12]. Plant-based medicines are famous for their low toxicity, minimal side effects, and high availability. The plants have potential medicinal uses due to their bioactive secondary metabolites, including the flavonoids [13–15]. Multiple studies have reported the benefits of flavonoids in overcoming various infectious and degenerative diseases [16–19].

*Garcinia* is a plant genus that is known to produce flavonoids in major quantities [20]. In Gayo Lues, a regency in Aceh Province, Indonesia, the local people have used *Garcinia macrophylla* Mart. stem barks as traditional medicine to combat the bacteria and free radicals. *G. macrophylla* had been studied for its pharmacology on inflammation, analgesic, microbial infection, and cancer [21]. However, the flavonoids in the *G. macrophylla* have not been thoroughly discussed. To the best of the authors' knowledge, this is the first report on the antibacterial, antioxidant, and anti-type 2 diabetic activities of the new biflavonoid extracted from *G. macrophylla* (Clusiaceae).

## 2. Materials and Methods

### 2.1. Materials

The instruments used in this research included rotary evaporator (Heidolph Laborota 4003 Control), UV-Vis (Shimadzu), FTIR (Prestige 21 Shimadzu), autoclave (Llenado-Filling), NMR (Agilent 500 MHz), shaker (Edmund Buhler D-7454 Bodelshausen), spectrophotometer (Spectro 20 D Plus Spectrophotometer), incubator (Lab-Line Instruments Ultra-Clean “100”), MultiCheck Nesco (Nesco Medlab, Indonesia), biological microscope (MEIJI TECHNO), Leica 2235 rotary microtome, electrothermal paraffin section mounting bath, SLEE paraffin embedding set, lamp ultraviolet (UV Laborgerate Vetter GMBH, Wiesloch), and MS (Shimadzu GCMS-QP 2010 Ultra).

The bacteria (*Escherichia coli* ATCC 25922 and *Staphylococcus aureus* ATCC 25923) were obtained from the Microbiology Laboratory, Faculty of Pharmacy at the University of Sumatera Utara (USU), Medan, Indonesia. The materials included in this research were Lactose Broth (LB), ascorbic acid 99.7%, distilled water, silica gel G 60 (Merck), Muller Hinton Agar (MHA), *n*-hexane 96%, dimethyl sulfoxide (DMSO) 99.9%, ethyl acetate 96%, NaCl, methanol 96%, dichloromethane 96%, paper disc (6 mm), cephazolin 30 *μ*g (disc 6 mm), strip test Nesco, metformin, glucose monohydrate p.a., Whatman filter paper grade 41, 2,2-diphenyl-1-picrylhydrazyl (DPPH), Neutral Buffered Formalin (NBF) 10%, ethanol (70%, 80%, 90%, 96%, and absolute), hematoxylin eosin, TLC preparative silica gel 60 F_254_, deuterated methanol (CD_3_OD), methanol 99.9%, and FeCl_3_.

### 2.2. Sample Collection

*G. macrophylla* stem bark sample was collected from Gayo Lues Regency, Aceh Province, Indonesia (4° 3′ 10,56″ N 97° 24′ 18,15″ E), in September 2016. It was identified by a botanist of Research Center for Biology at Indonesian Institute of Sciences (LIPI), Bogor, Indonesia. The specimen voucher was labeled as 5/Medang Kandis. In Indonesia, the plant is called *Kandis Gajah* [22].

### 2.3. Extraction, Isolation, and Elucidation

The sample was chopped and dried. 2.5 kg of the dried sample was extracted with maceration using methanol 96%. All filtrates obtained were concentrated with the rotary evaporator. The methanol extract (212.6 g) was sequentially partitioned with 96% *n*-hexane (500 mL) solvent and 96% ethyl acetate (1,500 mL) solvent in a separating funnel. The solvent of the fraction was evaporated to obtain the *n*-hexane fraction (10.5 g), the ethyl acetate fraction (101.4 g), and the methanol fraction (100.4 g), respectively.

An ethyl acetate extract fraction (50 g) was isolated with column chromatography (ф = 5 cm). Isolation was conducted using silica gel G 60 F (500 g) as stationary phase and dichloromethane-ethyl acetate as mobile phase, by the increase of polarity. The addition of polarity in the chromatography process was maintained until dichloromethane-ethyl acetate ratio reached 0:100 (v/v). Afterward, the purification to yield pure isolate was performed with rechromatography, preparative TLC, and recrystallization. A total of 60 mg of the pure isolate was elucidated by 1D-NMR (1H and 13C), 2D-NMR (HSQC and HMBC), GC-MS, FTIR, and UV-Vis spectrophotometers. The final product of this process was a biflavonoid compound **1**.

### 2.4. Antioxidant Assay

A free radical scavenging experiment was accomplished by modifying the DPPH reduction method [23]. This experiment was carried out using 99.9% methanol as a blank. DPPH 0.1 mM of 11 mL was prepared (0.0005 grams of DPPH crystal in 11 mL of 99.9% methanol solvent). A negative control was formulated by adding 3 mL of 99.9% methanol solvent to 1 mL of DPPH 0.1 mM. A positive control (ascorbic acid) and tested compound (compound **1**) were prepared in stock beforehand with concentrations of 100 ppm (0.001 grams in 10 mL of 99.9% methanol solvent), respectively. The positive control and the tested compound stock solutions were diluted to varying doses of 2, 4, 6, 8, and 10 (ppm), respectively. A total of 1 mL of 0.1 mM DPPH solution was pipetted and added to 3 mL variation of the positive control and the tested compound doses, respectively. All solutions were incubated under conditions at room temperature in a dark room for 30 minutes, and the process of measuring absorbance was continued at a wavelength of 517 nm with a spectrophotometer. All absorbance values were calculated for percent inhibition and IC_50_.

### 2.5. Antibacterial Assay

A subculture method was performed to inoculate each strain of bacteria [24]. A LB medium of 20 mL was prepared for each bacterium. A bacterium was taken from agar media stock and added to each LB medium. The bacterium in LB media was regenerated overnight at 37°C. The fresh bacterium was suspended in 10 mL NaCl 0.85% until an absorbance reached 0.5–0.8 (in order to afford 1 × 10^8^ CFU/mL) on OD_600_ by a spectrophotometer [25].

The antibacterial activities were carried out by the disc diffusion method [26]. 20 mL of MHA solution was poured into a Petri dish glass (size of 100 × 15 mm). Furthermore, fresh *E. coli* and *S. aureus* were smeared over agar media of the Petri dish. The paper discs containing the compound **1** (doses of 30 *μ*g/mL, 60 *μ*g/mL, and 120 *μ*g/mL), the positive control of cephazolin, and the negative control of DMSO were placed on the top of the growth media. Incubation was carried out at 37°C for 24 hours. The inhibition zones were measured by a caliper in millimeters (mm) as an indication of antibacterial activity.

### 2.6. Antidiabetic Assay

*Rattus norvegicus* rats (150–250 g) aged 2.5–3.0 months in healthy conditions were divided into 5 groups with 5 rats in each group. All rats were adapted for 7 days by giving pellets as food and distilled water as drinks in the cage [27]. Each rat for the experiment was given a group code, and its blood sugar level was checked initially after 7 days of adaptation (referred to as pretreatment). The group code was confirmed as follows: the negative control group was coded with B0, the positive control group was given the metformin drug coded with B1, and the groups were given the compound **1** with varying doses of 6, 7, and 8 (*μ*g/kg body weight) given their respective codes B2, B3, and B4. Furthermore, a glucose monohydrate was injected with a dose of 150 mg/kg body weight in all rats [28]. After 7 days of the glucose monohydrate administration, blood glucose levels were measured again in these rats (referred to as diabetic blood). A diabetic rat was given oral treatment in each group once a day, namely, group B0 (given the distilled water), B1 (given the metformin), B2 (given the compound **1** at dose of 6 *μ*g/kg body weight), B3 (given the compound **1** at dose of 7 *μ*g/kg body weight), and B4 (given the compound **1** at dose of 8 *μ*g/kg body weight). The blood glucose levels were checked every 7 days until they became normal again (referred to as posttreatment). Selected rats from each group at the end of the treatment were sacrificed for taking kidney, pancreatic, and liver organs in order to do histopathological analysis.

### 2.7. Histopathological Studies

The kidney, pancreas, and liver organs in the treatment rats (various group codes as B0, B1, B2, B3, and B4) and normal rats without the treatment (blank control with code A) were targeted for histopathological examination. The organs were soaked for 7 days into a 10% NBF before histopathology [29]. Histopathology was carried out by staining organs that have been sliced with hematoxylin-eosin dyes and placed on glass preparation. The glass preparation organs were observed using a biological microscope to determine cell performance in the kidneys, pancreas, and liver of rats.

### 2.8. Statistical Analysis

The obtained data were handled through Microsoft Excel 2010 software (Microsoft Corp., Redmond, WA). All data were presented as means ± standard deviation in the assay. The IC_50_ value in the antioxidant assay was calculated based on linear regression analysis of plot concentration variation (*μ*g/mL) to inhibition (%). Two-way ANOVA was used for the study of antidiabetic assay using GraphPad software Prism 7 (GraphPad Software, Inc., San Diego, California, USA). Matching between groups was carried out by Tukey's post hoc examination. *p* values <0.05 were considered significantly different. The one-way ANOVA for the study of histopathological data was run by R 3.6.2 software.

## 3. Results and Discussion

### 3.1. Structure Elucidation

The substance **1** isolated from the ethyl acetate fraction of the *G. macrophylla* stem barks possessed yellow solid appearance. UV spectrum gave the *λ*_max_ of MeOH at 260.0 nm and 274.0 nm, ascribed to the presence of *π*-*π*∗ electron excitation, which was typical for chromophore moiety with conjugated double bonding systems -C=C-C=C- on the aromatic rings. Meanwhile, the *λ*_max_ at 331.2 nm and 355.0 nm indicated the n-*π*∗ electron excitation (i.e., presence of heteroatom or conjugated carbonyl -C=C-C=O).

The FTIR spectrum of compound **1** which was subjected directly to the GC-MS instrument exhibited the specific absorption signals corresponding to particular functional groups at respective wavenumber; they were 3252-3387 cm^−1^ (–OH); 2924 cm^−1^ (C-H aliphatic), 1643 cm^−1^ and 1607 cm^−1^ (C=O), 1514 cm^−1^ and 1452 cm^−1^ (C=C aromatic); 1368 cm^−1^ and 1261 cm^−1^ (C-C aliphatic); and 1165 cm^−1^, 1088 cm^−1^, and 1049 cm^−1^ (C-O). Two fragmentations were present in GC-MS data, at m/*z* [M-H] 270 (C_15_H_11_O_5_) and 284 (C_15_H_8_O_6_).

The ^1^H-NMR spectrum of **1** suggested the presence of 13 protons: ten aromatic, two aliphatic, and one olefinic. The aromatic protons appeared at the *δ*_H_ (ppm) 5.96 (1H, d, *J* = 2.15 Hz), 5.98 (1H, d, *J* = 2.15 Hz), 7.07 (1H, d, *J* = 8.30 Hz), 6.64 (1H, d, *J* = 8.05 Hz), 6.43 (1H, d, *J* = 8.10 Hz), 7.10 (1H, d, *J* = 8.25 Hz), 6.25 (1H, s), 7.33 (1H, m), 6.91 (1H, d, *J* = 8.35 Hz), and 7.29 (1H, d, *J* = 8.40 Hz). Meanwhile, the aliphatic protons were characterized at the *δ*_H_ (ppm) 5.75 (1H, d, *J* = 11.90 Hz) and 4.83 (1H, d, *J* = 11.95 Hz), and the olefinic proton was detected at *δ*_H_ (ppm) 6.39 (1H, s).

The ^13^C-NMR spectrum of **1** displayed the 30 carbon atoms with the characteristics of two carbonyl carbons, 15 quaternary carbons, and 13 tertiary carbons. The carbonyl carbon signals were detected at the *δ*_C_ (ppm) 197.91 and 183.83. The signals of quaternary carbon appeared at the *δ*_C_ (ppm) 164.81, 165.73, 168.19, 103.19, 130.49, 158.81, 165.73, 157.37, 102.02, 163.31, 162.53, 104.95, 123.34, 146.79, and 150.94. The tertiary carbons were confirmed at the *δ*_C_ (ppm) 82.72, 50.84, 97.43, 96.42, 129.24, 115.61, 115.54, 129.24, 103.37, 99.83, 114.17, 116.84, and 120.55. Indeed, the DEPT data also confirmed the presence of tertiary and quaternary carbons.

**Figure 1:**
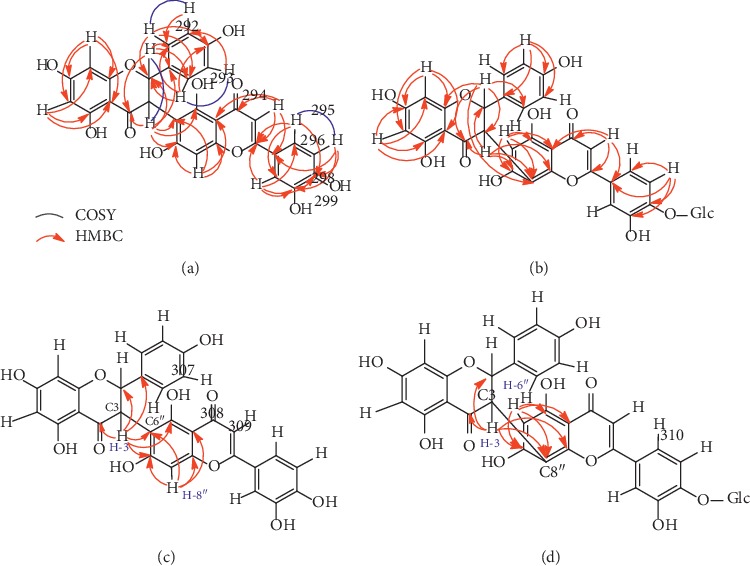
Comparison of HMBC correlation between **1** and **3** compounds. (a) Macrophylloflavone (**1**). (b) HMBC of morelloflavone-4‴-O-*β*-D-glucoside (**3**) [30]. (c) HMBC of 3-flavanone-6″-flavone. (d) HMBC of 3-flavanone-8″-flavone [30].

The HMBC cross-peak between protons and carbons demonstrated two aliphatic protons correlation to several carbons, i.e., H-2 (*δ*_H_ 5.75 ppm, 1H, d, *J* = 11.90 Hz) to C-4 (*δ*_C_ 197.91 ppm), C-1′ (*δ*_C_ 130.49 ppm), C-2′ (*δ*_C_ 129.24 ppm), C-6′ (*δ*_C_ 129.24 ppm); H-3 (*δ*_H_ 4.83 ppm, 1H, d, *J* = 11.95 Hz) to C-2 (*δ*_C_ 82.72 ppm), C-4 (*δ*_C_ 197.91 ppm), C-1′(*δ*_C_ 130.49 ppm), C-5″(*δ*_C_ 157.37 ppm), C-6″ (*δ*_C_ 102.02 ppm), C-7″ (*δ*_C_ 163.31 ppm). The olefinic H-3″ proton (*δ*_H_ 6.39 ppm, 1H, s) correlated to C-2″ (*δ*_C_ 165.73 ppm), C-4″ (*δ*_C_ 183.83 ppm), C-10″ (*δ*_C_ 104.95 ppm), C-1‴ (*δ*_C_ 123.34 ppm). The correspondence of ten aromatic protons is displayed in H-6 (*δ*_H_ 5.96 ppm, 1H, d, *J* = 2.15 Hz) to C-5 (*δ*_C_ 164.81 ppm), C-8 (*δ*_C_ 96.42 ppm), C-10 (*δ*_C_ 103.19 ppm); H-8 (*δ*_H_ 5.98 ppm, 1H, d, *J* = 2.15 Hz) to C-6 (*δ*_C_ 97.43 ppm), C-7 (*δ*_C_ 165.73 ppm), C-9 (*δ*_C_ 168.19 ppm), C-10 (*δ*_C_ 103.19 ppm); H-2′ (*δ*_H_ 7.07 ppm, 1H, d, *J* = 8.30 Hz) to C-2 (*δ*_C_ 82.72 ppm), C-4′ (*δ*_C_ 158.81 ppm), C-6′ (*δ*_C_ 129.24 ppm); H-3′ (*δ*_H_ 6.64 ppm, 1H, d, *J* = 8.05 Hz) to C-2′ (*δ*_C_ 129.24 ppm); H-5′ (*δ*_H_ 6.43 ppm, 1H, d, *J* = 8.10 Hz) to C-3′ (*δ*_C_ 115.61 ppm); H-6′ (*δ*_H_ 7.10 ppm, 1H, d, *J* = 8.25 Hz) to C-2 (*δ*_C_ 82.72 ppm), C-2′ (*δ*_C_ 129.24 ppm), C-4′ (*δ*_C_ 158.81 ppm); H-8″ (*δ*_H_ 6.25 ppm, 1H, s) to C-6″ (*δ*_C_ 102.02 ppm), C-7″ (*δ*_C_ 163.31 ppm), C-9″ (*δ*_C_ 162.53 ppm), C-10″ (*δ*_C_ 104.95 ppm); H-2‴ (*δ*_H_ 7.33 ppm, 1H, m) to C-2″ (*δ*_C_ 165.73 ppm), C-3‴ (*δ*_C_ 146.79 ppm), C-4‴ (*δ*_C_ 150.94 ppm), C-6‴ (*δ*_C_ 120.55 ppm); H-5‴(*δ*_H_ 6.91 ppm, 1H, d, *J* = 8.35 Hz) to C-1‴ (*δ*_C_ 123.34 ppm), C-3‴ (*δ*_C_ 146.79 ppm), C-4‴ (*δ*_C_ 150.94 ppm); and H-6‴ (*δ*_H_ 7.29 ppm, 1H, d, *J* = 8.40 Hz) to C-2″ (*δ*_C_ 165.73 ppm), C-2‴ (*δ*_C_ 114.17 ppm), C-4‴ (*δ*_C_ 150.94 ppm). The correlations between neighboring protons were indicated in the COSY spectrum data of H-2 with H-3; H-2′ with H-3′; H-5′ with H-6′; and 5‴ with 6‴. The two-dimensional correlation of the HMBC and the COSY cross-peaks is displayed in Figure 1.

**Figure 2:**
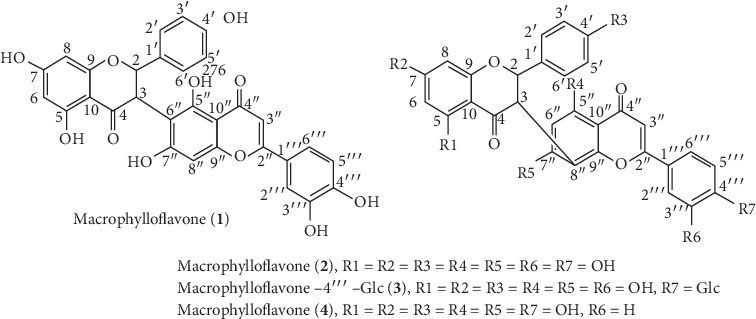
Macrophylloflavone (**1)**; morelloflavone (**2)** [37–42]; morelloflavone-4‴-O-*β*-D- glucoside (**3**) [30]; and volkensiflavone (**4**) [43].

Molecular assembly based on interpretation of 1D- and 2D-NMR data specified a flavanone skeletal pattern with naringenin feature [31–34] and a flavone skeletal pattern with luteolin feature [35, 36] constructing the compound **1** which is named macrophylloflavone. The molecular structure of **1** was also supported by its features based on the presence of chromophore in the UV spectra and the mass-to-charge ratio of FTIR. The NMR data for compound **1** were then confirmed by the NMR data from known compounds, i.e., morelloflavone [37–42], its glucoside derivative [30], and volkensiflavone [43], which have similar characteristics as shown in Tables 1 and 2. Analyses based on comparison with the spectroscopic data between compound **1** and a number of known related flavonoid dimers exhibited structural resemblance excluding for C-C linkage between flavanone-flavone moieties as shown in Figure 2.

The existence of the chemical shifts at *δ*_H_ (ppm) 5.75 (1H, d, *J* = 11.90 Hz, H-2), 4.83 (1H, d, *J* = 11.95 Hz, H-3), 5.96 (1H, d, *J* = 2.15 Hz, H-6), 5.98 (1H, d, *J* = 2.15 Hz, H-8), 7.07 (1H, d, *J* = 8.30 Hz, H-2′), 6.64 (1H, d, *J* = 8.05 Hz, H-3′), 6.43 (1H, d, *J* = 8.10 Hz, H-5′), and 7.10 (1H, d, *J* = 8.25 Hz, H-6′) exposed the same pattern as the naringenin compound [31–34]. The appearance of the signals at *δ*_H_ (ppm) 5.75 (1H, d, *J* = 11.90 Hz, H-2) and 4.83 (1H, d, *J* = 11.95 Hz, H-3) confirmed the *trans*-vicinal proton-proton relationship at the aliphatic carbon of the flavanone unit. The difference in chemical shift values between H-2 and H-3 was due to the electronegative influence of the oxygen atom in the sp^3^ hybridization carbon chain. H-2 was deshielded by adjacent oxygen atom and has a higher chemical shift than H-3 which was deshielded to a reduced magnitude of the carbonyl carbon [45]. In the meantime, the signals appeared at *δ*_H_ (ppm) 6.39 (1H, s, H-3 ″), 6.25 (1H, s, H-8″), 7.33 (1H, m, H-2‴), 6.91 (1H, d, *J* = 8.35 Hz, H-5‴), and 7.29 (1H, d, *J* = 8.40 Hz, H-6‴) confirmed the same pattern as the luteolin compound; also, the signal at *δ*_H_ (ppm) 6.39 (1H, s, H-3″) established the presence of a vinylic proton at flavone unit [35, 36]. In addition, such data was very much in accordance with the published data on the related biflavonoid compounds [30, 37–43].

The signals at *δ*_C-4″_ 183.83 ppm and *δ*_C-4_ 197.91 ppm were a unique attribute for carbonyl carbon at a dimeric flavonoid compound. Moreover, the signals at *δ*_C-2_ 82.72 ppm, *δ*_C-3_ 50.84 ppm, *δ*_C-6_ 97.43 ppm, *δ*_C-8_ 96.42 ppm, *δ*_C-3″_ 103.37 ppm, *δ*_C-6″_ 102.02 ppm, and *δ*_C-8″_ 99.83 ppm were a unique feature of the flavanone[3-6″] flavone skeleton pattern. It was clearly confirmed that the *δ*_C-6″_ appeared in the downfield region and the *δ*_C-8″_ existed in the upfield region. Referring to the *δ*_C-6″_ and *δ*_C-8″_ values of compounds **2**, **3**, and **4**, the *δ*_C-6″_ value always appeared in the upfield area compared to the *δ*_C-8″_ value which exists in the downfield area where the linkage occurs in the flavanone[3-8″] flavone skeleton pattern [30, 39–42, 44]. Linkage performance between C-3 in flavanone moiety and C-6″ in flavone moiety could also be observed through the HMBC relationship of compound **1** which was compared with the HMBC of compound **3** as shown in Figure 2. The overall assignment based on spectroscopic data of compound **1**, by comparing data to the model compounds, demonstrated that macrophylloflavone (**1**) is a new biflavonoid compound with linkage flavanone[3-6″] flavone system.

### 3.2. Antibacterial Activity

The classification of antimicrobial strength such as weak, moderate, and strong is indicated by the inhibition zones of <12, 12-20, and >20 mm, respectively [46]. Based on the results, the biflavonoid compound **1** was found to have strong antimicrobial activity against both the *E. coli* and the *S. aureus* bacteria. The inhibition zones in various concentrations were found to be around 16.65 ± 0.43 to 20.29 ± 0.28 mm (mean ± SD) for *E. coli* (30 *μ*g/mL, 60 *μ*g/mL, and 120 *μ*g/mL). Meanwhile, for *S. aureus*, the inhibition zone was around 15.54 ± 0.39 to 23.16 ± 0.32 mm (mean ± SD). The antibacterial properties of flavonoids were based on their ability to inhibit nucleic acid synthesis, cytoplasmic membrane function, energy metabolism, and porins in cell membranes [47].

### 3.3. Antioxidant Activity

The antioxidant performance of the compound **1** against DPPH free radicals gave the IC_50_ values of 3.69 ± 0.29 *μ*g/mL (mean ± SD). The strength of an antioxidant's activity is based on the IC_50_ value criteria; i.e., the IC_50_ value <10 *μ*g/mL indicates very strong activity, the value 10–50 *μ*g/mL suggests strong activity, the value 50–100 *μ*g/mL implies moderate activity, the value 100–250 *μ*g/mL reveals weak activity, and the value >250 *μ*g/mL signifies no activity [48]. In this research, the IC_50_ value of compound **1** was found to be <10 *μ*g/mL indicating a very strong antioxidant activity. Almost all flavonoid compounds are antioxidants. It is ascribed to their possession of hydroxyl groups in the flavonoids [49]. The compound **1** acted as an antioxidant agent donating its hydrogen atoms from the substituent content of the hydroxyl groups towards DPPH free radical, allowing the reduction of the DPPH radical. The reduction of DPPH radicals can be observed through the color change, from purple (DPPH) to yellow (1,1-diphenyl-2-picrylhydrazine) [50].

### 3.4. Anti-Type 2 Diabetes Mellitus Activity

**Figure 3:**
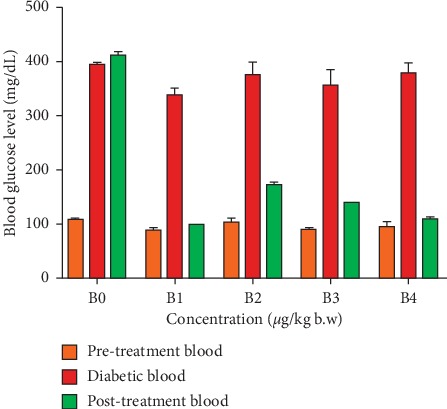
Histogram chart of the relationship of each group's treatment to changes in blood glucose levels.

The compound **1** has the ability to reduce the blood glucose levels in rats with diabetes (Figure 3). Several studies have reported that flavonoids contribute to the prevention of diabetes mellitus by regenerating the formation of *β*-cells pancreas which acts to produce insulin. The formation allows the decrease of glucose by converting it into energy, thus maintaining the blood glucose levels under a normal condition [51].

The results of the group treatment in decreasing the blood glucose levels are presented in Table 3 and Figure 3. The data were analyzed with two-way ANOVA to interpret the significance of the interaction effect at *F* (8, 40) = 146.2, *p* < 0.0001. The main effect between treatment (doses of compound **1**) and control (negative and positive controls) referred as *F* (2, 40) = 2985, *p* < 0.0001, produces a significantly different value in blood glucose levels at the posttreatment (mean ± SD), namely, negative control (412.20 ± 7.76), positive control (98.00 ± 1.67), dose 6 *μ*g/kg body weight (171.00 ± 3.81), dose 7 *μ*g/kg body weight (138.00 ± 1.87), and a dose of 8 *μ*g/kg body weight (108.40 ± 3.21). According to the American Diabetes Association, blood glucose levels >180 mg/dL indicate hyperglycemia, while the levels <100 mg/dL indicate hypoglycemia [52]. Therefore, it could be assumed that blood glucose levels given by the treatment group were found to be within the normal range. The blood glucose levels in the positive control group, however, indicate the risk of hypoglycemia. The blood glucose levels produced the main effect at F ratio of *F* (4, 20) = 239, *p* < 0.0001 generating the significant force. The analysis of Tukey's post hoc test showed that the positive control has a significant different effect of compound **1** on the dose of 6 *μ*g/kg body weight (*p* < 0.0001), 7 *μ*g/kg body weight (*p*=0.0049), and 8 *μ*g/kg body weight (*p*=0.0055). This means that all variations of compound **1** dosage have antidiabetic activity better than the positive control, but a very significant different result in reducing blood glucose in diabetic rats was shown by compound **1** at a dose of 6 *μ*g/kg body weight. In other words, there were variations in the high and low doses of compound **1** have no effect in decreasing the blood glucose of diabetic rats.

Decreasing of blood glucose levels in diabetic rats was caused by the activity of flavonoid dimers in compound **1**, i.e., naringenin and luteolin. Flavonoid compounds have double bonds, carbonyl groups, and hydroxy groups which are very important in the antidiabetic activity [53]. Naringenin and luteolin, in particular, have been reported to have the ability to overcome the hyperglycemia by inhibiting intestinal *α*-glucosidase activity, reducing oxidative stress, increasing antioxidant enzymes, and stimulating insulin secretion [54, 55]. The results of the decrease in blood sugar levels obtained after administration of compound **1** conclusively proved that the macrophylloflavone (**1**) has anti-type 2 diabetes property.

The histopathological data of kidney tubular cells, pancreatic *β*-cells, and liver hepatocytes cell required the normal rat cells (given code A), as a benchmark in the observations. The data in Table 4 have shown changes in the number of kidney tubular cells, pancreatic *β*-cells, and liver hepatocytes cells. These were ascribed to the presence of necrosis in cells by glucose monohydrate (alloxan). Alloxan can cause significant damage to these cells by triggering the formation of free radicals that lead to diabetes in rats [56].

**Figure 4:**
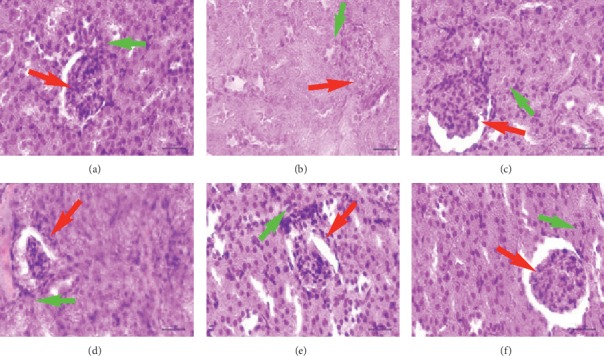
The kidney histology of rat at the blank control and various treatment groups. Red arrow: glomerulus; green arrow: proximal tubule. (a) A. (b) B0. (c) B1. (d) B2. (e) B3. (f) B4.

The administration of the compound **1** (on doses of B2, B3, and B4) reduced the necrosis in kidney tubular cells (Table 4). It was evident that the compound **1** could repair the tubular cell damage in the kidney. The number of necrotic cells in the kidney tubules in the treatment group with 8 *μ*g/kg body weight dose (B4) revealed the similarities with the normal rat kidney tubules (A) (*p* < 0.05). The kidneys contribute to glucose homeostasis through the processes of gluconeogenesis, glucose filtration, glucose reabsorption, and glucose consumption [57]. Kidney gluconeogenesis is more sensitive to insulin. The condition of diabetes results in an increase in kidney glucose production that is not proportional to the amount of insulin, hence the consequent reduction in glucose absorption in the proximal tubule by the insulin-independent process [58]. Excessive glucose and oxidative stress in the kidneys cause damage to tubular cells. The antioxidant properties of the compound **1** have produced a positive effect in preventing damage to kidney tubular cells so that the appearance of tubular cells is closer to the appearance of tubules under normal conditions (Figure 4).

**Figure 5:**
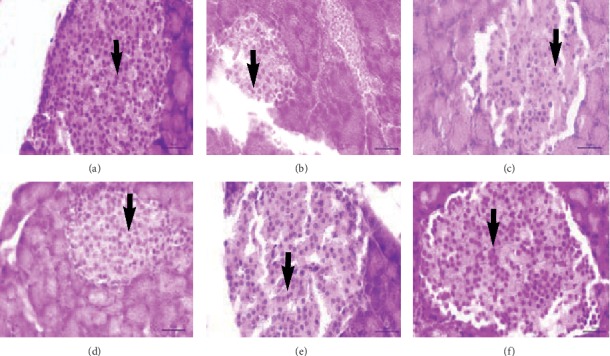
The pancreatic histology of rat at the blank control and various treatment groups. Black arrow: pancreatic beta cell. (a) A. (b) B0. (c) B1. (d) B2. (e) B3. (f) B4.

An increase in the number of pancreatic *β*-cells has occurred in the administration of the compound **1** at various doses (B2, B3, and B4) (Table 4). Administration of the compound **1** with a dose of 8 *μ*g/kg body weight (B4) has to afford no difference in the number of normal pancreatic *β*-cells (A) (*p* < 0.05). Application of the compound **1** was observed to increase the formation of *β*-cells in the pancreas (Figure 5). This was possible due to the compound **1** ability to neutralize free radicals that inhibit the formation of *β*-cells in the pancreas. Flavonoid compounds in the case of diabetes were used for maintaining the function of *β*-cell pancreatic and increasing the reformation of *β*-cells in the pancreas [59].

**Figure 6:**
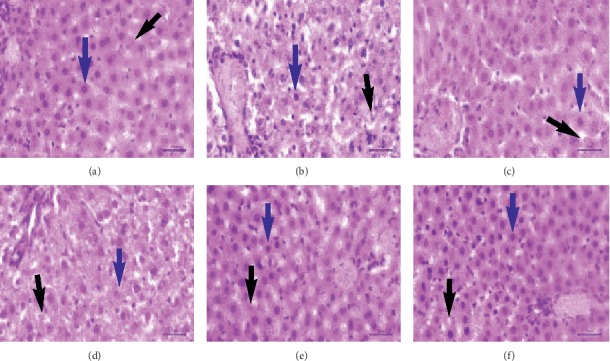
The liver histology of rat at the blank control and various treatment groups. Blue arrow: normal hepatocyte; black arrow: necrosis hepatocyte. (a) A. (b) B0. (c) B1. (d) B2. (e) B3. (f) B4.

The liver hepatocyte cell damage decreased after giving the compound **1** with various doses (B2, B3, and B4) (Table 4). The compound **1** with dose of 7 *μ*g/kg body weight (B3) and dose of 8 *μ*g/kg body weight (B4) resulted in a number of liver hepatocyte necrosis cells same as the number of normal hepatocyte necrosis cells (A) (*p* < 0.05). The compound **1** was shown not to damage liver hepatocyte cells (not toxic) and regenerate liver hepatocyte cells in the normal range (Figure 6). Antioxidant agents based on liver pathology are administrated to repair or prevent various liver diseases which are commonly caused by oxidative stress disorders [60].

## 4. Conclusions

The macrophylloflavone (**1**) as new biflavonoid compound with linkage 3-flavanone-6″-flavone has been successfully isolated from the ethyl acetate fraction from the stem bark of *G. macrophylla* plant. The *in vitro* evaluation of **1** suggested strong antibacterial and also antioxidant activities. More importantly, the *in vivo* assay exhibited the ability of **1** to decrease blood glucose levels in the diabetic rats to the normal level.

## Figures and Tables

**Table 1:** Comparison of ^1^H-NMR spectra in 3-flavanone-6″-flavone of macrophylloflavone (**1**) with a number of biflavonoid 3-flavanone-8″-flavone.

Compound	Position	2	3	4	5	6	7	8	9	10	1'	2'	3'	4'	5'	6'
Macrophylloflavone (**1**)	*δ*_H_ (ppm) (a)	5.75 (1H, d, *J* = 11.90 Hz)	4.83 (1H, d, *J* = 11.95 Hz)	—	—	5.96 (1H, d, *J* = 2.15 Hz)	—	5.98 (1H, d, *J* = 2.15 Hz)	—	—	—	7.07 (1H, d, *J* = 8.30 Hz)	6.64 (1H, d, *J* = 8.05 Hz)	—	6.43 (1H, d, *J* = 8.10 Hz)	7.10 (1H, d, *J* = 8.25 Hz)
Morelloflavone (**2**) [37]	*δ*_H_ (ppm) (b)	4.05 (1H, d, *J* = 12.00 Hz)	4.95 (1H, d, *J* = 12.00 Hz)	—	—	4.05 (1H, d)	—	4.05 (1H, d)	—	—	—	4.05 (1H, d)	4.05 (1H, d)	—	4.05 (1H, d)	4.05 (1H, d)
Morelloflavone (**2**) [38]	*δ*_H_ (ppm) (c)	5.87 (1H, d, *J* = 12.00 Hz)	5.07 (1H, d, *J* = 12.00 Hz)	—	—	6.10 (1H, d, *J* = 2.00 Hz)	—	6.38 (1H, d, *J* = 2.00 Hz)	—	—	—	7.30 (1H, d, *J* = 8.00 Hz)	6.63 (1H, d, *J* = 8.00 Hz)	—	6.63 (1H, d, *J* = 8.00 Hz)	7.30 (1H, d, *J* = 8.00 Hz)
Morelloflavone (**2**) [39]	*δ*_H_ (ppm) (d)	5.73 (1H, d, *J* = 12.00 Hz)	4.86 (1H, d, *J* = 12.00 Hz)	—	—	5.97 (1H, s)	—	5.97 (1H, s)	—	—	—	7.08 (1H, d, *J* = 9.00 Hz)	6.50 (1H, d, *J* = 9.00 Hz)	—	6.50 (1H, d, *J* = 9.00 Hz)	7.08 (1H, d, *J* = 9.00 Hz)
Morelloflavone (**2**) [40]	*δ*_H_ (ppm) (e)	5.64 (1H, d, *J* = 12.00 Hz)	4.83 (1H, d, *J* = 12.00 Hz)	—	—	5.95 (1H, s)	—	5.95 (1H, s)	—	—	—	7.09 (1H, d, *J* = 8.00 Hz)	6.50 (1H, d, *J* = 8.00 Hz)	—	6.50 (1H, d, *J* = 8.00 Hz)	7.09 (1H, d, *J* = 8.00 Hz)
Morelloflavone (**2**) [41]	*δ*_H_ (ppm) (f)	5.72 (1H, d, *J* = 12.00 Hz)	4.86 (1H, d, *J* = 12.00 Hz)	—	—	5.91 (1H, s)	—	5.91 (1H, s)	—	—	—	7.08 (1H, d, *J* = 8.00 Hz)	6.32 (1H, d, *J* = 8.00 Hz)	—	6.32 (1H, d, *J* = 8.00 Hz)	7.08 (1H, d, *J* = 8.00 Hz)
Morelloflavone (**2**) [42]	*δ*_H_ (ppm) (g)	5.88 (1H, d, *J* = 12.40 Hz)	5.00 (1H, d, *J* = 12.40 Hz)	—	—	6.04 (1H, br, s)	—	6.04 (1H, br, s)	—	—	—	7.25 (1H, d, *J* = 8.00 Hz)	6.54 (1H, d, *J* = 8.00 Hz)	—	6.54 (1H, d, *J* = 8.00 Hz)	7.25 (1H, d, *J* = 8.00 Hz)
Morelloflavone-4‴-O-*β*-D-glucoside (**3**) [30]	*δ*_H_ (ppm) (h)	5.89 (1H, d, *J* = 12.61 Hz)	4.93 (1H, d, *J* = 12.61 Hz)	—	—	5.94 (1H, d, *J* = 4.60 Hz)	—	6.53 (1H, d, *J* = 5.00 Hz)	—	—	—	6.63 (1H, dd, *J* = 7.60 Hz)	7.15 (1H, dd, *J* = 7.97 Hz)	—	7.15 (1H, dd, *J* = 7.97 Hz)	6.63 (1H, dd, *J* = 7.60 Hz)
Volkensiflavone (**4**) [43]	*δ*_H_ (ppm) (i)	4.30 (1H, d, *J* = 14.00 Hz)	5.10 (1H, d, *J* = 14.00 Hz)	—	—	3.9 (1H, s)	—	4.00 (1H, s)	—	—	—	2.90 (1H, d)	3.40 (1H, d)	—	3.40 (1H, d)	2.90 (1H, d)

Compound	Position	2″	3″	4″	5″	6″	7″	8″	9″	10″	1‴	2‴	3‴	4‴	5‴	6‴

Macrophylloflavone (**1**)	*δ*_H_ (ppm) (a)	—	6.39 (1H, s)	—	—	—	—	6.25 (1H, s)	—	—	—	7.33 (1H, m)	—	—	6.91 (1H, d, *J* = 8.35 Hz)	7.29 (1H, d, *J* = 8.40 Hz)
Morelloflavone (**2**) [37]	*δ*_H_ (ppm) (b)	—	3.90 (1H, s)	—	—	4.05 (1H, s)	—	—	—	—	—	4.95 (1H, d)	—	—	4.95 (1H, d)	4.95 (1H, d)
Morelloflavone (**2**) [38]	*δ*_H_ (ppm) (c)	—	6.55 (1H, s)	—	—	6.08 (1H, s)	—	—	—	—	—	7.52 (1H, m)	—	—	6.68 (1H, d, *J* = 8.00 Hz)	7.52 (1H, m)
Morelloflavone (**2**) [39]	*δ*_H_ (ppm) (d)	—	6.43 (1H, s)	—	—	6.20 (1H, s)	—	—	—	—	—	7.72 (1H, d, *J* = 2.00 Hz)	—	—	6.80 (1H, d, *J* = 9.00 Hz)	7.19 (1H, dd, *J* = 9.00, 2.00 Hz)
Morelloflavone (**2**) [40]	*δ*_H_ (ppm) (e)	—	6.44 (1H, s)	—	—	6.19 (1H, s)	—	—	—	—	—	7.37 (1H, m)	—	—	6.84 (1H, d, *J* = 8.00 Hz)	7.37 (1H, m)
Morelloflavone (**2**) [41]	*δ*_H_ (ppm) (f)	—	6.53 (1H, s)	—	—	6.17 (1H, s)	—	—	—	—	—	7.31 (1H, br, *s*)	—	—	7.20 (1H, br)	6.80 (1H, br)
Morelloflavone (**2**) [42]	*δ*_H_ (ppm) (g)	—	6.48 (1H, s)	—	—	6.32 (1H, s)	—	—	—	—	—	7.52 (1H, br, s)	—	—	7.03 (1H, d, *J* = 8.00 Hz)	7.54 (1H, br, d, *J* = 8.00 Hz)
Morelloflavone-4‴-O-*β*-D-glucoside (**3**) [30]	*δ*_H_ (ppm) (h)	—	6.76 (1H, s)	—	—	6.59 (1H, s)	—	—	—	—	—	7.32 (1H, s)	—	—	6.97 (1H, d, *J* = 8.10 Hz)	7.49 (1H, dd, *J* = 8.10 Hz)
Volkensiflavone (**4**) [43]	*δ*_H_ (ppm) (i)	—	3.45 (1H, s)	—	—	3.70 (1H, s)	—	—	—	—	—	2.40 (1H, d)	3.20 (1H, d)	—	3.20 (1H, d)	2.40 (1H, d)

Solvent and magnetic field strength for (a) CD_3_OD, 500 MHz; (b) acetone-*d*_6_; (c) DMSO-*d*_6_; (d) DMSO-*d*_6_, 100 MHz; (e) DMSO-*d*_6_; (f) DMSO-*d*_6_, 100 MHz; (g) CDCl_3_, 400 MHz; (h) DMSO-*d*_6_, 400 MHz; and (i) hexadeuteriodimethylsulphoxide, 100 MHz.

**Table 2:** Comparison of ^13^C-NMR spectra in 3-flavanone-6″-flavone of macrophylloflavone (**1**) with a number of biflavonoid 3-flavanone-8″-flavone.

Compound	Position	2	3	4	5	6	7	8	9	10	1′	2′	3′	4′	5′	6′
Macrophylloflavone (**1**)	*δ*_C_ (ppm) (a)	82.72	50.84	197.91	164.81	97.43	165.73	96.42	168.19	103.19	130.49	129.24	115.61	158.81	115.54	129.24
Morelloflavone (**2**) [44]	*δ*_C_ (ppm) (b)	81.00	48.70	195.60	163.50	96.20	166.30	95.20	162.50	101.50	128.00	128.10	114.40	157.10	114.40	128.10
Morelloflavone (**2**) [39]	*δ*_C_ (ppm) (c)	82.30	50.10	197.20	163.50	97.20	162.40	96.10	164.20	101.60	129.80	129.40	115.50	158.70	115.50	129.40
Morelloflavone (**2**) [40]	*δ*_C_ (ppm) (d)	81.00	48.40	196.30	161.80	95.40	163.60	96.30	166.60	101.60	128.20	128.60	114.50	157.40	114.50	128.60
Morelloflavone (**2**) [41]	*δ*_C_ (ppm) (e)	81.10	48.10	195.40	163.30	96.00	166.10	94.90	162.40	101.30	127.80	127.60	114.30	157.00	114.30	127.60
Morelloflavone (**2**) [42]	*δ*_C_ (ppm) (f)	81.50	49.20	196.50	164.70	96.30	166.40	95.20	164.00	102.10	129.20	128.40	114.60	157.50	114.60	128.40
Morelloflavone-4‴-O-*β*-D-glucoside (**3**) [30]	*δ*_C_ (ppm) (g)	82.60	49.10	198.00	162.00	98.10	164.10	97.60	166.00	102.80	129.80	128.60	115.70	158.70	115.70	128.60

Compound	Position	2″	3″	4″	5″	6″	7″	8″	9″	10″	1‴	2‴	3‴	4‴	5‴	6‴

Macrophylloflavone (**1**)	*δ*_C_ (ppm) (a)	165.73	103.37	183.83	157.37	102.02	163.31	99.83	162.53	104.95	123.34	114.17	146.79	150.94	116.84	120.55
Morelloflavone (**2**) [44]	*δ*_C_ (ppm) (b)	163.20	102.40	181.40	160.30	98.60	161.40	100.50	155.00	103.30	121.20	113.10	145.40	149.40	116.10	119.00
Morelloflavone (**2**) [39]	*δ*_C_ (ppm) (c)	164.20	103.80	183.10	164.90	99.60	165.60	103.90	165.80	103.00	123.50	114.20	146.30	150.00	116.50	120.70
Morelloflavone (**2**) [40]	*δ*_C_ (ppm) (d)	163.80	102.30	181.70	160.60	98.70	162.90	100.60	155.30	103.20	121.10	113.40	145.70	149.80	116.20	119.40
Morelloflavone (**2**) [41]	*δ*_C_ (ppm) (e)	163.30	102.60	181.00	160.10	98.30	161.50	99.30	154.80	102.60	121.30	113.30	145.30	149.20	115.60	118.40
Morelloflavone (**2**) [42]	*δ*_C_ (ppm) (f)	163.40	102.90	182.30	164.70	98.70	161.70	104.00	155.90	100.80	122.60	113.30	145.50	149.30	115.80	119.60
Morelloflavone-4‴-O-*β*-D-glucoside (**3**) [30]	*δ*_C_ (ppm) (g)	170.60	104.10	183.50	158.30	98.20	161.10	103.90	153.30	104.60	120.50	114.60	145.40	148.70	117.20	121.00

Solvent and magnetic field strength for (a) CD_3_OD, 125 MHz; (b) DMSO-*d*_6_, 22.64 MHz; (c) DMSO-*d*_6_, 25.10 MHz; (d) DMSO-*d*_6_; (e) DMSO-*d*_6_; (f) CDCl_3_; and (g) DMSO-*d*_6_, 100 MHz.

**Table 3:** The effect of compound **1** on blood glucose levels of diabetic rats.

No.	Treatment	Blood glucose levels (mean ± SD, mg/dL)		
		Pretreatment blood	Diabetic blood	Posttreatment blood
**1**	B0	105.00 ± 3.94	396.00 ± 3.67	412.20 ± 7.76
	B1	88.20 ± 4.17	339.20 ± 12.49	98.00 ± 1.67
	B2	103.40 ± 6.80	377.00 ± 25.33	171.00 ± 3.81
	B3	87.00 ± 4.42	357.40 ± 28.87	138.00 ± 1.87
	B4	93.80 ± 11.12	379.40 ± 21.33	108.40 ± 3.21

**2**	ANOVA table	F (DFn, DFd)	*p* value	
	Interaction	*F* (8, 40) = 146.2	<0.0001	
	Dose (biflavonoid and control)	*F* (2, 40) = 2985	<0.0001	
	Blood glucose level	*F* (4, 20) = 239	<0.0001	

**3**	Tukey's post hoc test	*p* value	Significant?	
	B0 versus B1	<0.0001	Yes	
	B1 versus B2	<0.0001	Yes	
	B1 versus B3	0.0049	Yes	
	B1 versus B4	0.0055	Yes	

Different letters indicate significant differences between the groups (*p* < 0.05). Tukey's post hoc test following the two-way ANOVA. B0: the negative control, B1: the positive control, B2: the compound **1** given at a dose of 6 *μ*g/kg body weight, B3: the compound **1** given at a dose of 7 *μ*g/kg body weight, B4: the compound **1** given at a dose of 8 *μ*g/kg body weight.

**Table 4:** A total number of cells on histology of kidney, pancreas, and liver of rats in various groups.

Treatment	Histology of rat kidney		Histology of rat pancreas	Histology of rat liver	
	Normal cells of tubules (cells ± SD)	Necrosis cells of tubules (cells ± SD)	Beta cells of pancreatic (cells ± SD)	Normal cells of hepatocyte (cells ± SD)	Necrosis cells of hepatocyte (cells ± SD)
A	382 ± 8.72^a^	103 ± 12.49^e^	286 ± 13.00^a^	298 ± 8.19^a^	75 ± 9.17^d^
B0	299 ± 13.53^c^	162 ± 4.36^a^	184 ± 8.00^d^	262 ± 13.08^c^	113 ± 6.56^a^
B1	328 ± 17.35^b^	136 ± 7.94^bc^	252 ± 7.81^c^	281 ± 9.54^ab^	91 ± 4.58^bc^
B2	285 ± 15.09^c^	154 ± 14.00^ab^	241 ± 7.21^c^	276 ± 10.00^bc^	102 ± 12.17^ab^
B3	343 ± 5.29^b^	127 ± 6.24^cd^	269 ± 9.64^b^	284 ± 7.81^ab^	86 ± 6.08^cd^
B4	374 ± 10.44^a^	112 ± 14.93^de^	280 ± 7.00^ab^	287 ± 4.36^ab^	78 ± 8.54^cd^

A: the blank control, B0: the negative control, B1: the positive control, B2: the compound **1** given at a dose of 6 *μ*g/kg body weight, B3: the compound **1** given at a dose of 7 *μ*g/kg body weight, B4: the compound **1** given at a dose of 8 *μ*g/kg body weight. Different letters showed significant differences between the groups (*p* < 0.05).

## Data Availability

The data generated to support the findings during this study are included within this article.
